# Adolescent Health Literacy and Neighbourhood Features: HBSC Findings from Czech Republic, Poland, and Slovakia

**DOI:** 10.3390/ijerph18147388

**Published:** 2021-07-10

**Authors:** Dorota Kleszczewska, Katarzyna Porwit, Zuzana Boberova, Eric Sigmund, Jana Vasickova, Leena Paakkari

**Affiliations:** 1 Institute of Mother and Child Foundation, 01-211 Warsaw, Poland; 2 Centre of Migration Research, University of Warsaw, 02-093 Warsaw, Poland; katarzyna.porwit@uw.edu.pl; 3 Institute of Biology and Ecology, Faculty of Science, Pavol Jozef Šafárik University in Košice, Mánesova 23, 040-01 Košice, Slovakia; zuzana.boberova@upjs.sk; 4 Faculty of Physical Culture, Palacký University Olomouc, 771 47 Olomouc, Czech Republic; erik.sigmund@upol.cz; 5 Department of Social Sciences in Kinanthropology, Faculty of Physical Culture, 771 11 Olomouc, Czech Republic; jana.vasickova@upol.cz; 6 Research Center for Health Promotion, Faculty of Sport and Health Sciences, University of Jyväskylä, 40014 Jyväskylä, Finland; leena.paakkari@jyu.fi

**Keywords:** health literacy, adolescents, neighbourhood

## Abstract

The role of supportive environments on health, wellbeing, and longevity has been widely recognized. However, there is no strong empirical evidence on the association between health literacy (HL) as a particular health-related competence and neighbourhoods. Therefore, the aim of the study was to assess the association between the features of neighbourhoods and the level of HL competencies of young people from three countries (Czech Republic, Poland, Slovakia). Self-reported data from an international sample of 11,521 students aged 13–15 years participating in the Health Behaviour in School-aged Children Study (HBSC) in the year 2018 were included in the analyses. The level of HL shows a strong positive relationship with family wealth, and a significant relationship is maintained in all studied countries. Both social and structural features of neighbourhoods turned out to have an impact on students’ HL. However, HL is most clearly explained by the school environment. This study confirms the school effect on higher levels of HL competences in adolescents. This indicates the need to invest in schools located in less affluent areas to generally improve the level of education, implement modern health education combined with HL, and strengthen the social and health competencies of students.

## 1. Introduction

The role of supportive environments in health, wellbeing, and longevity has been widely recognised. According to the Ottawa Charter for Health Promotion [1], health is created within daily settings where people “learn, work, play, and love”. The settings can be places or social contexts “in which people engage in daily activities in which environmental, organizational, and personal factors interact to affect health and wellbeing” [2,3]. 

The role of the environment on life, health, and development of man has been discussed in the literature for many years. One of the strongest voices in it is Urie Brofenbrenner’s ecological theory of systems, which has been the theoretical basis of many studies [4]. It assumes that the development of a man, and in particular of a child, consists in the formation of relationships between the individual and the closer and more distant environment. Man affects the environment, and the environment determines development, including human health. This model was the basis for a research model of the Health Behaviour in School-aged Children (HBSC) network, which for years has been a unique source of knowledge about the lifestyle that shapes adolescent health [5]. In this paper, the presented analyses are based on data collected during the last round of HBSC research.

The neighbourhood is an important setting for adolescents’ development, along with family, school, and various social networks [6,7].

Neighbourhoods have been linked with adolescents’ competence development, though it is not easy to estimate the impact of the neighbourhood and that of, for instance, schools, due to their overlapping demographics [8]. Thus far, the empirical evidence on the association between health literacy (HL) as a particular health-related competence and neighbourhood is lacking. However, studies on several cognitive skills have shown that gaps in skills development grow especially during summer (non-school) periods, highlighting the role of disadvantaged experiences faced in neighbourhoods and homes in shaping inequalities [9,10,11]. The meta-analysis of Niewenhuis and Hooimeijer [8] showed that “the relation between neighbourhoods and individual educational outcomes is a function of neighbourhood poverty, the neighbourhood’s educational climate, the proportion of ethnic/migrant groups, and social disorganisation in the neighbourhood”. Furthermore, the level of affluence of the living environment is more than the level of poverty or deprivation [12], is often linked to the neighbourhood social capital, and they both correlate with educational outcomes [13], but also with various health outcomes, including life expectancy, mental health problems and self-rated health [14,15].

It is also worth stressing that the influence of neighbourhood on individuals might depend on and change with age, being stronger for adolescents [8]. For younger children, the influence of the family on health is more pronounced [16], and experiences from childhood may affect health later in life [17]. Adolescence is a time when non-family environments start to affect young people’s health, and emotional and behavioural problems may occur or worsen. Adolescents are socialised not only by their parents but also by the various adults and peers they interact with [18]. 

Neighbourhood covers many assets that impact adolescents’ empowerment [19]. Oliva et al., while concerning “security, availability of structured extracurricular activities or positive reinforcement and allocation of responsibilities and roles for adolescents in the community” refer to “neighbourhood developmental assets”, and they find many similarities between these assets and social capital [19]. Indeed, the social cohesion, shared norms, and capacity of neighbourhood residents to solve problems and share information are determinants that may impact health and development [20]. The concept of social capital, understood as social bonds and active engagement, is relational and based on the resources people can access through others. Social capital has been strongly linked to health, and it is argued that through social cohesion and access to resources, individuals’ health is affected by their social network [21]. On the other hand, HL as a set of “personal knowledge and competencies which accumulate through daily activities, social interactions and across generations”, and which is “mediated by cultural and situational demands that are placed on people, organizations and society” [22], is an important capacity “to gain access to and use neighbourhood resources to benefit health” [23]. Similarly, in addition to promoting and maintaining one’s own health, HL is an ability to “access, understand, appraise, and use information and services” contributing to the promotion of health and wellbeing in our close surroundings [24].

HL is a dynamic quality going beyond the individual level, where strong community health literacy diminishes the likelihood of anyone being left behind because of their individual level of HL. However, what is health literacy in the first place? The Ratzan and Parker [25] definition that was included in the Institute of Medicine (IOM) has been widely used in the literature. They have defined health literacy as: “The degree to which individuals have the capacity to obtain, process, and understand basic health information and services needed to make appropriate health decisions.

Community HL comprises the assets and capacities within communities, such as cities, neighbourhoods, or groups, that promote health for all the community’s members [26]. Community seems to be even more important if we assume that HL can be developed and improved through organised school health education, and that the environment can be modified in ways that make it easier for young people to obtain, understand, and use information that promotes and maintains health [27]. Higgins et al. [28] revealed the presence of external influences in the community and societal context that can impact how adolescents experience health education in forming their HL. They identified the potential components reflecting the socioeconomic status of the neighbouring community, such as availability of fast-food outlets and presence/quality of in-school cafeteria facilities and vending machine options, levels and volume of nearby traffic and walkability of the school community. Additionally, Paakkari et al. [29] identified sport clubs’ settings as an important moderator for HL. HL has also been identified as a key component of health-promoting schools [30] and the concept of health literate schools was introduced [31]. However, there is almost no empirical evidence about the association between the features of neighbourhoods and the level of HL among adolescents. The authors of this research assume that higher levels of HL positively impact the health of young people in society, and it is important to understand what the relationships between the features of neighbourhoods and the level of HL among adolescents are [30].

Understanding those relations can be especially important if we take into consideration the inequalities in health and attempts at addressing them [31]. Furthermore, discussions on the associations between HL and contextual factors are underdeveloped [32].

Therefore, the aim of the study was to assess the association between the features of neighbourhoods and the level of HL competencies (Health Literacy for School-Aged Children—HLSAC-index) of young people from three CEE countries (Czech Republic, Poland, Slovakia). Additionally, the similarities and differences between countries were investigated. 

## 2. Materials and Methods

### 2.1. Participants

Self-reported data from an international sample of 11,521 students from CEE, aged 12.6 to 16.3 years (mean age 14.44 ± 1.04 years) and participating in the Health Behaviour in School-aged Children Study (HBSC) study in the 2017–2018 school year were included in the analyses. The Czech sample was the most numerous (54.4% of the respondents). In all three countries, data came from representative nationwide surveys; the number of participating schools randomly selected for the study were: 227 (Czech Republic), 174 (Poland), and 108 (Slovakia). In the combined sample, 48.6% of representatives were boys and 51.4% were girls. The gender structure of the respondents was similar in all three countries (*p* = 0.125). Surveyed adolescents belonged to two age groups (13- and 15-year olds), rigorously released according to HBSC research network protocol (95% of the sample must be in the age defined category). The age structure of the Polish and Czech trials was similar, while the Slovak trials were relatively younger. The data chosen for further analysis had to contain complete information about neighbourhood features and included answers to at least seven questions about literacy health. Detailed data on the number of students from individual countries qualified for the analysis are presented in Table 1. The procedure for conducting the research was the same in all three countries and in line with the HBSC protocol. The differences related to the survey method occurred in the form of data collection. In Czech Republic and Slovakia it was an online questionnaire, while in Poland a traditional paper one was filled out.

### 2.2. Health Literacy

A package of questions concerning HL is optional in the HBSC research protocol. The youth was asked to respond to 10 statements, having a choice of four categories of answers, from not at all true to absolutely true. In the sample of 11,521 students from three countries, the general index, known in the literature as HLSAC, had a single-factor structure, and the reliability of the scale was 0.886. The psychometric properties of the HLSAC scale have been described in previous publications, based on results from 10 countries, including the three included in this research [33]. 10,814 respondents answered all 10 questions. In the remaining 707 cases, an approximate value converted into a range of 10–40 points was estimated, allowing one (600 cases), two (84), or three (23) missing data.

### 2.3. Neighbourhood 

The neighbourhood package is also optional in HBSC research protocols. Czech Republic, Poland, and Slovakia are the only countries in the HBSC network that included both optional packages in the last research round: on HLSAC and on the neighbourhood. At the first stage, the authors conducted a simple analysis of three social and two structural features of the neighbourhood. All five of these determinants have been coded into three intervals. In the case of two indexes, a conventional division was made that 50–60% were a mean value range and 20–25% are extreme. The distribution of answers to the questions is presented in Table 2.

When describing social features, young people assessed the level of safety in the area and responded to the statement that it was a good place to live. The third variable was social capital index, ranging from 0–16 points. The index was created with four categories of answers, and it has already been described in the literature [34]. 

A low level of funding is 0–8 points, and a high level was 14–16 points. In the studied sample of 11,521 students from 3 countries, this index had a single factor; the reliability of the scale was 0.739.

Describing the structural features of the environment, adolescents described how affluent their neighbourhood was and tried to assess the level of its deprivation on the basis of three features. The summary scale of perception ranged from 0–6 points. A score of 0–2 points was considered a high level of deprivation, and a score of 5–6 points a low one. In merged data from the three countries this index was a single-factorial one, and the reliability of the scale is 0.695.

At the stage of complex analyses the authors limited analysis to one collective index for assessing social characteristics of the neighbourhood. It was built on the basis of six original questions. It was an index standardised as z-score (mean 0 and SD = 1 in the entire international sample), estimated using the PCA (principal component analysis) method with reliability at the level of 0.738. The z-score index was adopted as a variable characterising the structural features.

Additional features (apart from the country of residence) were gender, age, and family affluence, measured with the Family Affluence Scale (FAS) described in the literature. The FAS scale consisted of six questions and had a range of 0–13 points. The mean FAS rating in the combined sample from the three countries was 8.00 ± 2.37. Adolescents from Poland and Slovakia assessed the affluence of their families on approximately the same level (7.82 ± 2.33 and 7.82 ± 2.43, respectively), while the families of Czech students turned out to be the richest (8.14 ± 2.36).

### 2.4. Statistical Methods

The HLSAC index was used as the main dependent variable; its distribution was examined using the Kolmogorov–Smirnov test with the Lilliefors correction. The results showed that this distribution differed significantly from the normal distribution (*p* < 0.001), so in further analyses non-parametric tests and generalised models resistant to atypical distributions of variables were used. The differentiation between schools in the ICC (intraclass coefficient) were estimated separately for each country from the so-called null multilevel model, treating school as a random factor.

In simple comparisons, the chi-square test was used to test the relationship of categorised features (country vs. neighbourhood features), the non-parametric Kruskal–Wallis test for comparisons of HL indexes between three countries, and the non-parametric Mann–Whitney test for comparisons between two age groups and between boys and girls. With regard to the KW test, pairs of countries were additionally compared as a non-parametric variant of post hoc analysis. 

In the multivariate analyses, only collective indicators of the structural and social features of the neighbourhood were taken into account, building one z-score indicator for each of these features. It has an average value of 0 and a standard deviation of 1 in the international sample, as described previously in z-scores.

Generalised linear models (GLMZ) with main effects and 2nd degree interaction were used in multivariate analysis. A graphic illustration of selected interactions is presented based on the general linear model adjusted for other factors. The analyses are complemented by the country-specific GLMZ models presented in the Table S1 as additional electronic material.

## 3. Results 

### 3.1. Level of HLSAC

In the study group of 11,521 students from three countries, the mean HLSAC index was 30.39 (SD = 5.41). Girls achieved higher values than boys (30.57 ± 5.09 vs. 30.19 ± 5.72; *p* = 0.001). There were no differences between the two age groups (*p* = 0.920). The mean HLSAC was similar in Poland and Czech Republic (30.45 ± 4.52 vs. 30.14 ± 5.86; *p* = 0.369 in post hoc pairwise comparison), while it was higher in Slovakia (31.11 ± 5.19) than in the other two countries (*p* < 0.001). Poland was the only country with significant differences in HLSAC levels between boys and girls, in favour of the latter. The differences between age groups appeared in Poland, in favour of older students. HLSAC levels showed a strong positive relationship with family wealth as measured by the FAS scale, and a significant relationship was maintained in all countries. The Spearman correlation between FAS and HLSAC was at rho = 0.099 (*p* < 0.001). The rho coefficients were 0.096, 0.116, and 0.107 in Czech Republic, Poland, and Slovakia, respectively. 

### 3.2. Neighbourhood

A comparison of the countries in terms of the three social and two structural neighbourhood characteristics is shown in Table 2. The percentage of students who were unsure about their safety in their place of living was low, and more than half of the respondents (53.5%) felt always safe. The percentage of students who always felt safe was lowest in Poland and highest in Slovakia. Polish adolescents were less likely than their peers in the other two countries to describe their neighbourhood as a good place to live. The inference about differences between countries in the perception of social features of the neighbourhood changes with respect to the social capital index. Significant differences between countries still persist, but Slovak students were the least advantaged. The distribution of responses to the question on the subjective assessment of the neighbourhood’s socio-economic status shows some disturbing differences, which may also be due to the way the question was phrased in the different language versions. In Czech Republic, the highest percentage of negative evaluation was found, while in Slovakia the percentage of positive evaluation was incomparably high. Considering the categorised index of perception of problems in the local area, the worst results in Poland were noted. 

After generating overall neighbourhood indexes standardised as z-scores, it was shown that the averages for individual countries differ. The index describing social features was equal to: Czech Republic: 0.05 ± 1.02; Poland: −0.06 ± 0.98; Slovakia: −0.06 ± 0.97, respectively. In the case of the index assessing the structural features of the neighbourhood, the lowest score was obtained in Poland (−0.35 ± 1.00) and a clearly outlying high score in Slovakia (0.42 ± 1.02), while a positive value slightly higher than zero was found in Czech Republic (0.06 ± 0.93).

Table 3 presents the mean HLSAC indices according to the social characteristics of the neighbourhoods of participants. A statistically significant relationship was shown for all three variables in each participating country. In all three countries, HL increases most when the level of social capital is high.

In the case of structural characteristics, only Poland showed no relationship between HLSAC level and perception of local problems.

In the case of structural characteristics, only Poland showed no relationship between HLSAC level and perception of local problems. The average HL increases to 31.7 if the structural features are assessed positively. This effect is the most visible in Slovakia (Table 4).

### 3.3. School Effect

School diversity was examined in terms of the variables under study, including HLSAC level, FAS, and social and structural characteristics of the neighbourhood (Table 5). Very unfavourable results were achieved by schools in which a small number of students were examined. The extremely low results should therefore be viewed with caution. In all countries, in the best schools the average HLSAC score exceeded 34 points. According to ICC, the school factor explained between 1.81–3.56% of the variation in this index, most notably in Poland. In terms of the average level of FAS, the mean values ranged from 3.00 to 11.71 FAS points on the school level. The three countries analysed differed markedly on the ICC coefficient. The school effect was least pronounced in Czech Republic and most pronounced in Poland. For the standardised index of social and structural neighbourhood characteristics, the school effect was most visible in Czech Republic.

### 3.4. Multifactorial Analysis

A series of multifactorial generalized linear models (GMLZ) with HLSAC index as a dependent variable were analysed. Gender, age (continuous), country, and continuous indices related to family wealth and neighbourhood status were included as independent variables (Table 6). The main effects and all possible two-way interactions were examined. A model containing all main effects and two two-way interactions was found to be optimal. The two-way interaction between country and neighbourhood structural characteristics and between social and structural characteristics proved to be significant. After including these interactions, the main effect of neighbourhood features remains significant. 

**Figure 1:**
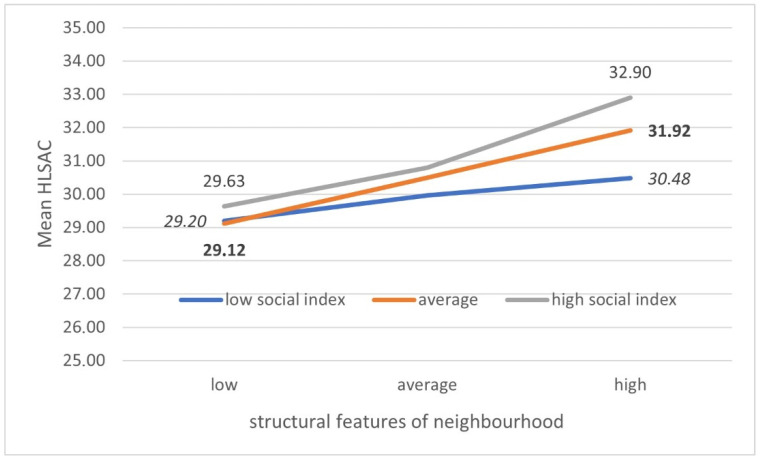
Average health literacy in school-aged children (HLSAC) level by social and structural neighbourhood feature (adjusted for gender, age, country, and family affluence (FAS)).

**Figure 2:**
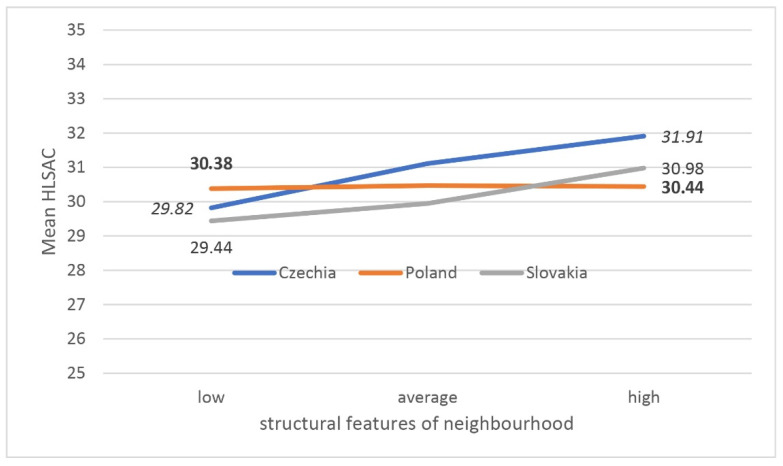
Average health literacy in school aged children (HLSAC) level by country and structural neighbourhood features (adjusted for gender, age, social features, and family affluence (FAS)).

Figure 1 and Figure 2 illustrate the disclosed interactions. The z-score indices describing the surroundings were conventionally divided into three ranges, where the extreme ones cover 20% of the international sample values. 

First, the interaction between social and structural features (Figure 1) turned out to be significant.

In the case of a rather low rating of social features, the changes associated with improvements in structural features are small. In the case of a rather good rating of social characteristics, the average HLSAC index increases from 30.48 in highly deprived areas to 32.90 in the most affluent ones.

This proves that the abovementioned two types of environmental features have a cumulative protective effect. The second significant interaction concerns the comparison of the relationship with the structural features of the neighbourhood between the countries (Figure 2).

Changes associated with improvements in structural characteristics are associated with an improvement in the HLSAC index of 2.09 points in Czech Republic and 1.54 points in Slovakia, while in Poland there is a minimal difference with no upward trend.

Country-specific models can complement the analyses (Table S1). The main effect of gender, age, and social characteristics of family and environment, and three possible two-way interactions between social indices, were included in the models. The association with gender was maintained in all countries, with the HLSAC index worsening in boys compared to girls. The association with age was only seen in Poland. A strong positive association with FAS also emerged in all three countries. The association with neighbourhood social characteristics was significant in all three countries but weakest in Slovakia (*p* = 0.043). The association with neighbourhood structural characteristics was significant only in Czech Republic. The interaction between social and structural neighbourhood characteristics was found to be significant in Czech Republic and Poland. There was no significant interaction between social or structural neighbourhood characteristics and FAS as predictors of HLSAC. Only in Poland was the interaction of FAS with structural features on the borderline of statistical significance (*p* = 0.056). This means that in Poland, as the structural characteristics of the environment improve, HLSAC increases only in affluent families. In poor families, students’ HL competences even worsen in more advantaged areas compared to more deprived ones.

## 4. Discussion

When discussing the results of our analyses, it should be noted that they are in line with a wide range of research on the association between the features of environment and health. It is also another contribution to the discussion on including the HL-related question module in the multi-threaded HBSC questionnaire. 

However, we should first of all refer to the results confirmed by this study regarding differences between participating countries, as well as the differences between the schools surveyed in these countries.

The obtained results confirmed that the HLSAC scale is a valuable research tool, enriching the HBSC research questionnaire, which can be recommended for use in many countries. Using the example of three Central European countries, the validity of combining the HL results with other optional packages available in the HBSC study protocol was demonstrated. In the conceptual model of HBSC research based on Bronfenrenner’s ecological systems theory [4], both HL and neighbourhood [35] were taken into account. Our study is the first to validate this part of the theoretical model from an international perspective.

The results of this study confirmed an existing association between the level of adolescents’ HL from three Central European countries (CEE countries) and neighbourhood features: where young people live is important to the HL competences they have. This paper can be an important contribution to the evidence base on HL and to project HL programmes and interventions targeted at adolescents—if they are meant to be effective, they should take into account the background that young people grow up in. This is in line with the extensive systematic review on HL in childhood and youth analysed by Broder et al. [36]. They have come to the same conclusions, namely, that the community where children and adolescents live may have an impact on their HL competences. 

A significant interaction was also demonstrated between the social and structural characteristics of the environment and the level of the HLSAC index. In highly deprived areas, higher social capital does not improve HL levels. Only the accumulation of the positive impact of high levels of both environmental features is visible. Some studies indicate that in poor areas there may also be a high level of social bonds, which translates into the strengthening of selected health competences, such as self-efficacy [37].

In this study HL among youth differs due to age and gender. Girls achieved higher values of HL than boys, and this association is maintained in all represented countries, and was also confirmed in previous studies [38]. Following the literature, it could be explained by the fact that girls are more interested in education in general [39], succeed better in schools [40], and are often better educated in adulthood [8,14,41] The association with age was only observed in Poland, where older children achieved better HL levels. This difference was also found in previous research by Paakari et al. where the HL level was lower for boys than for girls, and lower for 7th graders than for 9th graders [42,43] This is an interesting area for further research because in literature this relationship is not widely confirmed—the results where age does not affect the HL level of adolescents are in the majority [44,45].

A strong positive association with family affluence emerged in adolescents from all three participating countries. These findings can be confirmed elsewhere [46,47]. Children from higher status households showed better health knowledge. One explanation for this may be the difficulty less-educated parents have in providing relevant health-related knowledge to their children [48,49,50], and in this case the inequality in health is passed to the next generation.

A significant relationship between the level of HL and the social features of the neighbourhood was shown in adolescents from all three CEE countries. Social features were described in the study as a safe place to live, good place to live and through the social capital of the environment. Remarkably, in Poland, as the structural characteristics of the environment improve, the level of HL increases only in affluent families, while in poor families it gets even worse. This is of particular importance when we bear in mind that HL can be a key determinant of health and is strongly related to health inequalities that are more visible in more stratified societies.

Lithuanian researchers have analysed the effect of socioeconomic status on families and adolescents’ level of HL. In that study, almost 40% of the families were categorised as having low affluence. That is why in the CEE countries where the affluence of societies is still low it is even more important to study the moderating role of a family’s socioeconomic status on children’s HL [51]. In the study called *Well-Being of Adolescents in Vulnerable Environments* (WAVE), authors compared the perceptions of neighbourhood factors among 2320 adolescents aged 15–19 years and examined the associations between factors within the physical and social environments. Mmari et al., like the authors of this study, confirmed that the perceptions about the physical and social environments within a neighbourhood are important to study among adolescents living in disadvantaged communities [52].

This study confirms the school effect on level of health competences in adolescents. In the literature, school-based interventions promoting HL have been studied and found to be effective. International bodies recommend incorporation of health-related tasks into school lessons and consider teaching young people a good investment for the future. Schools can empower youths by promoting HL [53], can provide an excellent opportunity and critical facilities in which many agencies can work together for the betterment of the youth, and can play a facilitative role to bring the societal components together in which children act as members of families, peers, schools, and communities [54]. 

CEE countries are less affected by inequalities in health related to ethnic origin or the status of immigrants (the problem is still the outflow of people to other countries [55]), but they are affected by inequalities related to low family well-being and uneven development of regions. Studies in adult population in Hungary on HL revealed that socio-economic status had a strong influence on HL level [56] and that the local deprivation affects academic performance [57]. 

The idea of education for health literacy is being implemented in most developed countries with a generally high level of health education. Based on data from three CEE countries, it would be advisable to include a broader environmental context to such activities [58].

## 5. Strengths and Weaknesses of the Study 

One of the strengths of this study is that it seems to be the first to validate this part of the theoretical model (relation between neighbourhood’s features and the HL level of adolescents) from an international perspective.

Furthermore, the study is limited only to three countries. As noted earlier, only these countries included the neighbourhood question module in the HBSC survey in their last round simultaneously with the HLSAC. The strong advantage of the study is a large and homogeneous sample of students in terms of age (*N* = 11,521) and the fact that the selected countries come from one geographical region in Central Europe, have a similar languages, and have a lot in common in the history of the last few decades. Language issues seem to be important here and to affect the comparability of the results in inter-country analyses, despite efforts to professionally translate the questionnaire in all countries carrying out HBSC research. Due to the selection of countries, this study fits perfectly with the subject of this Special Issue, devoted to the health of adolescents from this part of Europe.

The limitation of this study is its sectional character, which does not entitle us to infer a cause-and-effect relationship. A number of variables describing the environment in the place of residence were included in the analyses, on the basis of which general indicators (measures) referring separately to social and structural characteristics were built. The analyses were adjusted for the wealth of the family. A limitation may be not having included the additional variables that, in the light of available literature, may correlate with the level of HL. These could be a general health assessment or the level of academic achievement, which may be a measure of general cognitive abilities. However, this type of question was excluded from the HBSC protocol in the 2017/18 round.

## 6. Conclusions

The study has proven that there is a need for further analysis of the relation between HL and neighbourhood features due to obtained differences in the results between three countries from the same region. 

The results of our research, in particular the part concerning the school environment, show the greatest diversity of schools in terms of family wealth, but also, for example, in Czech Republic, schools turned out to be clearly differentiated in terms of the structural features of the environment. 

This indicates the need to invest in schools located in less affluent areas in order to generally improve the level of education, implement modern health education combined with the construction of HL, and strengthen the social and health competences of students. 

It seems that the need to implement school programs is recognized in the three analysed countries. Intervention and community programs seem to be less developed. Less is also said about the importance of leadership and advocacy.

## Figures and Tables

**Table 1:** Sample characteristics (*N* = 11,521).

	Czech Republic	Poland	Slovakia
Total	6281	3341	1899
Gender			
Boys	3106	1593	899
Girls	3175	1748	1000
Age			
13 years	3052	1620	1078
15 years	3229	1721	821

**Table 2:** Samples by neighbourhood features (%).

	Czech Republic	Poland	Slovakia	*p*
Social				
Safe place				
Sometimes or rarely/never	7.3	10.1	8.2	Chi-sq = 421.3
Most of time	35.6	50.3	26.1	df = 4
Always	57.1	39.6	65.8	*p* < 0.001
Good place to live				
Not good	4.7	5.1	4.2	Chi-sq = 229.5
Ok	22.1	34.7	19.8	df = 4
Good or very good	73.2	60.3	76.0	*p* < 0.001
Social capital index				
Low	25.7	25.6	31.2	Chi-sq = 49.7
Average	53.7	56.2	54.1	df = 4
High	20.6	18.2	14.6	*p* < 0.001
Structural				
Area well off				
Not well off	17.1	5.1	5.9	Chi-sq = 2372.8
Average	62.8	73.2	21.6	df = 4
Very well off	20.1	21.8	72.5	*p* < 0.001
Local area problems				
Poor (high deprivation)	16.7	35.9	13.4	Chi-sq = 665.8
Average	49.0	43.3	44.0	df = 4
Good (low deprivation)	34.2	20.8	42.5	*p* < 0.001

**Table 3:** Mean health literacy by neighbourhood social features mean ± SD.

	Czech Republic	Poland	Slovakia
Safe place			
Sometimes or rarely/never	27.7 ± 6.9	29.6 ± 4.8	28.1 ± 6.4
Most of time	29.6 ± 5.4	30.3 ± 4.3	30.0 ± 4.8
Always	30.8 ± 5.9	30.9 ± 4.7	31.9 ± 5.0
K-W chi-sq	156.9	30.5	98.3
*p*	<0.001	<0.001	<0.001
Good place to live			
Not good	28.3 ± 7.1	30.3 ± 4.8	28.3 ± 6.8
Ok	29.1 ± 5.7	29.8 ± 4.3	30.0 ± 5.6
Good or very good	30.6 ± 5.8	30.8 ± 4.6	31.6 ± 4.9
K-W chi-sq	101.4	44.8	36.8
*p*	<0.001	<0.001	<0.001
Social capital index			
Low	29.1 ± 6.0	29.6 ± 4.6	30.0 ± 5.5
Average	30.1 ± 5.4	30.4 ± 4.3	31.3 ± 4.6
High	31.6 ± 6.6	31.7 ± 4.7	32.7 ± 6.0
K-W chi-sq	168.6	81.5	64.0
*p*	<0.001	<0.001	<0.001

**Table 4:** Mean health literacy by neighbourhood structural features.

	Czech Republic	Poland	Slovakia
Area well off			
Not well off	29.0 ± 6.4	29.1 ± 5.3	28.7 ± 7.0
Average	30.2 ± 5.5	30.3 ± 4.3	29.9 ± 5.3
Very well off	31.1 ± 6.4	31.2 ± 4.9	31.7 ± 4.9
K-W chi-sq	84.5	37.8	47.5
*p*	<0.001	<0.001	<0.001
Local area problems			
Poor (high deprivation)	29.3 ± 6.8	30.4 ± 4.6	29.9 ± 6.2
Average	29.9 ± 5.5	30.4 ± 4.4	30.9 ± 5.0
Good (low deprivation)	30.9 ± 5.7	30.6 ± 4.7	31.7 ± 5.0
K-W chi-sq	64.9	0.7	20.0
*p*	<0.001	0.688	<0.001

**Table 5:** Differences between schools in terms of HLSAC level and social indices.

Combined Index	Czech Republic		Poland		Slovakia	
	Range of School Mean Value	ICC (%)	Range of School Mean Value	ICC (%)	Range of School Mean Value	ICC (%)
HLSAC index (10–40)	22.00–34.74	2.10	26.36–34.50	3.56	24.04–34.67	1.81
FAS (0–13)	4.00–10.03	6.94	4.57–11.71	16.35	3.00–10.18	9.34
Social features of neighbourhood (z-score)	−1.20–0.66	5.73	−0.89–0.92	2.19	−0.86–0.61	3.41
Structural features of neighbourhood (z-score)	−1.82–0.71	6.86	−1.19–0.98	2.79	−0.85–1.05	2.47

ICC—intraclass correlation, HLSAC—health literacy in school aged children, FAS—family affluence scale.

**Table 6:** Generalised linear model (GLMZ) for HLSAC index.

Independent Variable	B	SE	Wald Chi-Sq	Df	*p*
Constant	27.805	0.7241	1474.531	1	0.000
Gender (female ref.)	−0.682	0.0995	46.998	1	0.000
2. Age (cont.)	0.141	0.0480	8.646	1	0.003
3. FAS (0–13)	0.180	0.0211	73.190	1	0.000
4. Country (Slovakia ref.)					
Czech Republic	−0.994	0.1491	44.486	1	0.000
Poland	−0.545	0.1650	10.904	1	0.001
Social features (z-score)	0.988	0.0536	339.840	1	0.000
6. Structural features (z-score)	0.541	0.1208	20.018	1	0.000
7 5 × 6	0.308	0.0445	48.024	1	0.000
8. 4 × 6 (Slovakia ref.)					
Czech Republic	−0.145	0.1398	1.076	1	0.300
Poland	−0.598	0.1509	15.718	1	0.000
(Scale)	27.339	0.3640			

HLSAC—health literacy for school-aged children, FAS—family affluence scale.

## Data Availability

Data available on request.

## Supplementary Materials

The following are available online at https://www.mdpi.com/article/10.3390/ijerph18147388/s1, Table S1: Country specific GLMZ models.
