# Supplementary material for: Adolescent Health Literacy and Neighbourhood Features: HBSC Findings from Czech Republic, Poland, and Slovakia
Source: Int J Environ Res Public Health. 2021 Jul 10;18(14):7388. doi: 10.3390/ijerph18147388 (PMC8303563; doi:10.3390/ijerph18147388)
Supplement: Supplementary file 1 [file ijerph-18-07388-s001.zip › ijerph-1223705-supplementary.pdf]

International Journal of
Environmental Research and Public Health

MDPI

Table S1. Country specific GLMZ models.

Variable	Czechia			Poland			Slovakia		
	**B**	**SE**	**p**	**B**	**SE**	**p**	**B**	**SE**	**p**
Const.	28.633	1.051	0	23.085	1.099	0	29.75	1.83	0
1. Gender (female ref.)	−0.543	0.147	0	−0.934	0.154	0	−0.687	0.232	0.003
2. Age	0.02	0.07	0.777	0.42	0.074	0	0	0.122	0.998
3. FAS (0-13)	0.159	0.031	0	0.216	0.035	0	0.204	0.052	0
4. Social features (z-score)	0.912	0.262	0	1.175	0.28	0	0.829	0.409	0.043
5. Structural features (z-score)	0.829	0.295	0.005	−0.524	0.275	0.057	0.531	0.383	0.166
2-way interactions									
3*4	0.008	0.031	0.79	−0.032	0.034	0.348	0.041	0.05	0.407
3*5	−0.051	0.034	0.14	0.063	0.033	0.056	−0.01	0.047	0.826
4*5	0.446	0.068	0	0.177	0.074	0.016	0.06	0.098	0.539
(Scale)	32.524	0.585		19.036	0.471		24.138	0.797	
